# Water-Soluble Starch-Based Copolymers Synthesized by Electron Beam Irradiation: Physicochemical and Functional Characterization

**DOI:** 10.3390/ma15031061

**Published:** 2022-01-29

**Authors:** Monica R. Nemțanu, Mirela Brașoveanu, Elena Pincu, Viorica Meltzer

**Affiliations:** 1 Electron Accelerators Laboratory, National Institute for Lasers, Plasma and Radiation Physics, 409 Atomiştilor St., P.O. Box MG-36, 077125 Magurele, Romania; monica.nemtanu@inflpr.ro; 2 Department of Physical Chemistry, Faculty of Chemistry, University of Bucharest, 4-12 Regina Elisabeta Bd., 030018 Bucharest, Romania; elena.pincu@chimie.unibuc.ro (E.P.); meltzerviorica@yahoo.com (V.M.)

**Keywords:** polysaccharide, radiation, grafting copolymerization, intrinsic viscosity, thermal behavior, flocculant

## Abstract

Modification of natural polymers for applications in the treatment of waste and surface waters is a continuous concern of researchers and technologists in close relation to the advantages they provide as related to classical polymeric flocculants. In this work, copolymers of starch-graft-polyacrylamide (St-*g*-PAM) were synthesized by electron beam irradiation used as the free radical initiator by applying different irradiation doses and dose rates. St-*g*-PAM loaded with ex situ prepared silver nanoparticles was also synthesized by using an accelerated electron beam. The graft copolymers were characterized by chemical analysis, rheology, and differential scanning calorimetry (DSC). The results showed that the level of grafting (monomer conversion coefficient and residual monomer concentration), intrinsic viscosity and thermal behavior (thermodynamic parameters) were influenced by the irradiation dose, dose rate and presence of silver nanoparticles. The flocculation performances of the synthesized copolymers were also tested on water from the meat industry in experiments at the laboratory level. In the coagulation–flocculation process, the copolymer aqueous solutions showed good efficiency to improve different water quality indicators.

## 1. Introduction

Renewable raw materials are increasingly used nowadays due to both the rapid development of society in recent decades and the growing interest in using natural, biodegradable and affordable resources. As an example, the modification of natural polymers to be used in various applications, such as flocculants for wastewater treatment [1,2,3], matrices for controlled drug release [4,5], rheological modifiers for different applications [6,7] is an ongoing concern of scientists.

Organic flocculants, typically polymeric in nature, are widely used in the modern processes of potable water and wastewater treatment [8,9,10,11]. They can be traditionally synthesized by chemical reactions [3,12,13] controlled by catalysts and reaction conditions. Instead, the use of irradiation techniques (gamma radiation or electron beams) allows high efficiency and control of various processes (i.e., grafting, cross-linking) involved in the synthesis of flocculants, avoiding the use of chemical initiators/catalysts [12,14,15,16] or heating processes, which are undesirable in such syntheses.

Grafting is an effective way of regulating the properties of biopolymers, which can be “tailor-made” according to the needs and produce highly efficient graft copolymers usable as flocculants [17,18]. Cross-linking is another type of reaction that can lead to modification and functionalization of natural polymers by increasing their molecular weight and consequently improving and extending their functionality as flocculants [18]. However, the most used method for the synthesis of biopolymer-based flocculants is graft copolymerization because the chemical combination of organic synthetic polymer with natural polymer produces organic, natural polymer hybrid materials with desirable properties of both components [19]. Thus, a graft copolymer combines the best properties of both components and has unique properties compared to the original components.

Synthetic polymers based on vinyl monomers, such as acrylic acid and acrylamide, with flocculating features are effective and relatively cheap, with wide applications due to their varied qualities, but being poorly biodegradable and harmful to the environment [20]. They are often used in the copolymerization reaction with natural materials such as polysaccharides (i.e., starch, cellulose, guar gum, sodium alginate, etc.) and proteins (soy protein) in order to produce eco-friendly products [20,21]. Moreover, to improve their functionality with antimicrobial features, the biopolymer-based flocculants can be enriched with metal nanoparticles. For instance, silver nanoparticles (AgNPs) are considered as a non-toxic and environmentally friendly material with significant inhibitory effects against microbial pathogens [20,22,23], being lately incorporated in different polymer matrices as an agent with antimicrobial activity [24,25,26]. AgNPs can be synthesized by chemical (reduction of silver ions with a chemical reducing agent), physical (i.e., sputter deposition, laser ablation, irradiation, etc.) and biological methods [23,27,28]. Therefore, organic polymeric flocculants are truly confirmed materials for water treatment, and when improved with silver nanoparticles, they have the advantage that they can simultaneously have an effect on microbial loading. There is a special interest both in the methods of flocculant production and in the direction of flocculant materials based on natural polymers and improved with silver nanoparticles.

Electron beam (e-beam) irradiation is an environmentally friendly practice for the synthesis of advanced materials with unique properties. E-beam-induced grafting is a rapid technique that can be performed without an initiator or other chemical catalysts, resulting in low byproduct levels and hazards [29]. Moreover, e-beam processing involves low costs due to the indirect operating costs and the irradiation dose that depends on the power of the beam and its efficiency of use, as well as the factor of converting the beam power from the consumed power of the facility [30]. Scarce information is available about starch grafting by e-beam irradiation to produce flocculating materials. However, our previous investigations [31,32,33,34] on starch grafting by e-beam irradiation showed that starch-graft copolymers with flocculating abilities can be rapidly produced by simultaneous electron beam irradiation with low doses and no additional chemicals.

The present work deals with the physicochemical and functional characterization of starch-based copolymers and starch-based copolymer-loaded silver nanoparticles synthesized by using electron beam irradiation. Therefore, the purpose of this study was to point out relevant aspects related to new flocculating materials that can be a starting point for a new generation of products with unique functional properties, low toxicity and biodegradable, designed to be effective in environmental protection. At the same time, we aimed to give a deeper insight into a practical and ecological method of radiation-based synthesis for flocculants functionalized with metallic nanoparticles that meet the quality claims, which is able to replace the classical methods involving pollution risks, complex procedures and high costs.

## 2. Materials and Methods

### 2.1. Materials

Unmodified regular corn starch containing approximately 73% amylopectin and 27% amylose (S4126; white powder;) was purchased from Sigma-Aldrich (St. Louis, MO, USA), and acrylamide (A17157; 98+%; white; crystalline) was purchased from Alfa Aesar (Karlsruhe, Germany). Other chemicals were of analytical grade and purchased from SC Chimreactiv SRL (Bucuresti, Romania). Table 1 explicitly shows the materials used in the preparation of copolymers and their properties.

### 2.2. Synthesis of Starch-Graft Acrylamide Copolymers

Graft copolymers were synthesized in two steps: (1) preparation of solutions containing starch and monomer; (2) irradiation of solutions by using electron beam.

In the first step, starch samples were prepared by gelatinizing powder starch in distilled water. Acrylamide and sodium chloride (7.5%) was added to starch sample with further stirring, resulting in a starch: acrylamide (St:AMD weight ratio = 1:10) homogenous samples. Sodium chloride is one of the critical parameters in electron beam-induced polymerization, and the additional use of NaCl in the range of 5% to 10% can increase the monomer conversion (over 80%) and level of intrinsic viscosity of copolymers, reducing also residual monomer [35,36]. Moreover, the addition of NaCl to the chemical composition of the monomer solution allows the reduction of the irradiation dose which further leads to an increase in the electron beam efficiency, namely the initiation rate along with the fast consumption of the free radicals in the presence of sodium ions [36]. Silver nanoparticles (80 μmol/L) prepared ex situ according to Nemţanu et al. [37] were added to one of the samples.

Therefore, the homogenous samples resulting from the first stage were then subjected to e-beam irradiation. The sample processing was performed in static mode by using an accelerated e-beam provided by a linear accelerator ALID-7 of energy of 5.5 MeV (NILPRP, Bucharest-Măgurele, Romania). The electron accelerator installation is of traveling-wave type, using microwaves in the S-band at 2.99 GHz that propagate in a disk-loaded tube of about 2 m long; the microwaves are produced by an EEV-M5125 type magnetron that provides 2 MW of power in pulses of 4 μs [38]. The ALID-7 accelerator is used in different experimental researches in the field of radiation [21,39,40,41]. The sample irradiations were carried out with different irradiation doses (D = 0.7–1.2 kGy) and dose rates ($\dot{D}$ = 0.5–0.7 kGy/min) as shown in Table 2, at the room temperature (25 ± 1 °C) and ambient pressure under air. The configuration of the irradiation field at the distance of 52 cm from accelerator exit window where the samples to be irradiated were placed as well as the irradiation doses were checked by using aqueous chemical dosimetry, which involved ferrous sulfate solutions (Fricke and super-Fricke dosimetry) [42,43,44,45]. The irradiation parameters for this study were selected based on our previous study on optimization of the modification of starch by electron beam grafting for the synthesis of water-soluble copolymers [32,46]. In addition, we decided that the high dose rates can cause high radical density, leading to rapid generation of homopolymer in the case of acrylamide [47], which was undesirable for the present investigation.

**Scheme 1:**
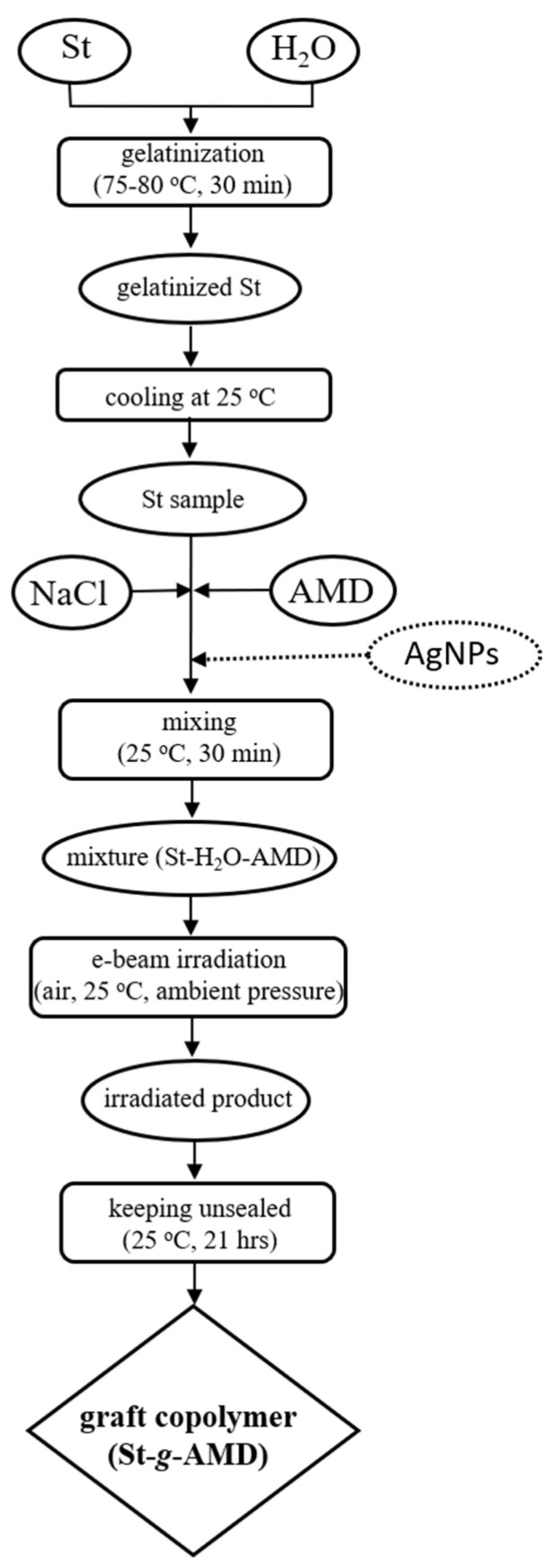
Synthesis of graft copolymers by using electron beam irradiation.

Scheme 1 shows the stages of graft copolymer synthesis by simultaneous graft copolymerization using electron beam irradiation.

### 2.3. Characterization of Graft Copolymers

The physicochemical properties of synthesized copolymers were evaluated through chemical analysis and rheology, while the functionality as flocculant agents was tested on wastewater from the meat industry.

#### 2.3.1. Level of Grafting

The monomer conversion coefficient (*C_c_*) and the unreacted monomer (*R_m_*) were evaluated by determination of the vinyl monomer double bonds before and after copolymerization. Thus, the copolymer solution was prepared by dissolving graft copolymer (2 g) in distilled water (200 mL) stored overnight at room temperature (25 ± 1 °C), and further, the obtained solution was magnetically stirred (300 rpm) in order to obtain a homogenous solution. Distilled water (100 mL) and 4 mol/L sodium chloride solution (100 mL) were added to this solution. Volume of 25 mL of copolymer solution was then transferred to a 250 mL Erlenmeyer flask and 10 mL of bromide–bromate solution (containing 35 g of potassium bromide and 2.783 g of potassium bromate per liter) and 2.5 mL of hydrochloric acid (18.5%) were added; then the flask was well stoppered. The solution was left to stand in the dark for 20 min and shaken frequently. After this time, 2.5 mL of 20% potassium iodide was rapidly added to flask content and the excess of the iodine liberated was titrated with 0.1 mol/L sodium thiosulfate (Na_2_S_2_O_3_) solution using 1 mL starch indicator solution (1%). A blank determination was made as well.

The residual monomer (*R_m_*) and the conversion coefficient (*C_c_*) were calculated from the following equations:

(1) $$R_{m}=0.0568\times \frac{V_{b}-V_{g}}{W_{g}}\times 100$$

where *R_m_* is the content of residual monomer (g of unreacted monomer/100 g copolymer), 0.0568 is a constant expressed in g/mL, *V_b_* is the volume of Na_2_S_2_O_3_ used for titration of the blank sample (mL), *V_g_* is the volume of Na_2_S_2_O_3_ used for titration of graft copolymer sample (mL), *W_g_* is the weight of the graft copolymer (g),

and

(2) $$C_{c}=\frac{M_{i}-R_{m}}{M_{i}}\times 100$$

where *C_c_* is the coefficient of the monomer conversion (g of reacted monomer/100 g copolymer), *M_i_* is the monomer content in sample before irradiation (g of monomer/100 g sample), *R_m_* is the content of residual monomer (g of unreacted monomer/100 g copolymer).

#### 2.3.2. Intrinsic Viscosity

The intrinsic viscosity [*η*] was calculated using the extrapolation method. The viscosity was carried out on diluted copolymer solutions (<1%) using 1 mol/L sodium chloride solution as a solvent. The prepared solutions were measured with a Hoppler type BH-2 viscometer at 25 °C, and the intrinsic viscosity was then determined by extrapolating the linear curve of the reduced viscosities that were calculated as a function of the selected concentrations.

#### 2.3.3. Differential Scanning Calorimetry (DSC)

DSC measurements were performed with a differential scanning calorimeter Perkin Elmer Diamond (Waltham, MA, USA) under argon atmosphere. The apparatus was calibrated with high purity indium as a standard substance. Samples of 1–5 mg were weighed with a Partner XA analytical balance (Radwag, Radom, Poland), with a precision of 10 μg. The samples sealed in aluminum crucibles were then heated in the temperature range 298–625 K at a heating rate of 10 K/min.

### 2.4. Flocculation Study

The graft copolymers were evaluated for their flocculation abilities in coagulation–flocculation experiments on wastewater collected from a meat processing plant. The load of this type of water is strongly influenced by the type of production and facilities, and this water proves a high strength in terms of biochemical oxygen demand, chemical oxygen demand, suspended solids, fatty matters (fat, oil and grease), nitrogen and phosphorus, compared to domestic waters [48]. In the herein investigation, the copolymer flocculation abilities were assessed in coagulation–flocculation experiments at the laboratory level, using inorganic coagulants (600 mg/L CaCO_3_ and 600 mg/L Al_2_(SO_4_)_3_) and a dosage of 8 mg/L of 0.2% aqueous solution flocculant (graft copolymer).

Quality parameters such as pH, total suspended solids (*TSS*), fatty matters (*FM*), chemical oxygen demand (*COD*), and biochemical oxygen demand (*BOD_5_*) were evaluated according to standardized methods [49,50,51,52] in order to look over the effect of the polymer addition on the degree of purification of the tested water. For each quality parameter, the flocculation efficiency (*FE*) was calculated based on the formula:

(3) $$FE\%=\frac{C_{0}-C}{C_{0}}\times 100$$

where *C*_0_ and *C* are the concentrations (in mg/L) of the investigated parameter before and after the tested water treatment.

### 2.5. Statistics

The results obtained and reported in this paper are expressed as mean values±standard deviation of triplicate determinations, except for the flocculation study. Experimental data were processed using OriginPro 8.1 (OriginLab Corporation, Northampton, MA, USA, 2016), Microsoft^®^ Excel 2010 (Microsoft Corporation, Redmond, WA, USA), and InfoStat versión 2018 [53]. Data were statistically analyzed by using analysis of variance with Fisher LSD (least significant differences) post-hoc test to discern the statistical difference. A probability value *p* ≤ 0.05 was considered statistically significant.

## 3. Results

Graft copolymers, varying the irradiation dose D and dose rate $\dot{D}$ and maintaining constant the concentrations of monomer and polysaccharide were synthesized. The synthesized copolymers were evaluated from a physicochemical and functional point of view. The characterization through chemical analysis and rheology was performed to point out the grafting level and viscosity, while the functionality as flocculant agents was tested on surface water from the meat industry.

### 3.1. Level of Grafting

A high conversion coefficient reflects a high efficiency of monomer transformation in the polymerization process and a considerable reduction of residual monomer content. Some characteristics of the graft copolymers synthesized under electron beam field by varying the irradiation dose D (free radical generator) and dose rate $\dot{D}\;$ are displayed in Table 3.

The monomer conversion coefficient *C_c_* exceeded 90% of all samples, with most values being about 96%. The results indicated that the highest value of the conversion coefficient (96.6 ± 0.5%) was obtained by using an irradiation dose of 1 kGy at a constant dose rate of 0.7 kGy/min (*Batch 1*). This finding is in agreement with Biswal et al. [47] who showed that grafting levels of ~100% could also be achieved at a dose of ~0.8 kGy at any of the dose rates up to 5 kGy/h in the case of the radiation-induced grafting of acrylamide onto guar gum in order to synthesis new copolymers with flocculating properties for industrial effluents.

The variation of dose rate by using a constant irradiation dose of 1 kGy (*Batch 2*) led to a slight decrease (*p* ≤ 0.05) of the conversion coefficient with the decrease in the dose rate. Consequently, the residual monomer *R_m_* followed the variation of the conversion coefficient. Thus, the copolymers synthesized at an irradiation dose of 1 kGy and dose rates of 0.6 and 0.7 kGy/min, respectively, had the lowest values of the residual monomer content. Moreover, the conversion coefficient and residual monomer were much better correlated with dose rate than irradiation dose. Thus, *C_c_* and *R_m_* showed strong correlations with dose rate (*r* = 0.991 and *r* = −0.993, respectively) and good correlations with irradiation dose (*r* = 0.789 and *r* ~−0.805, respectively).

**Scheme 2:**
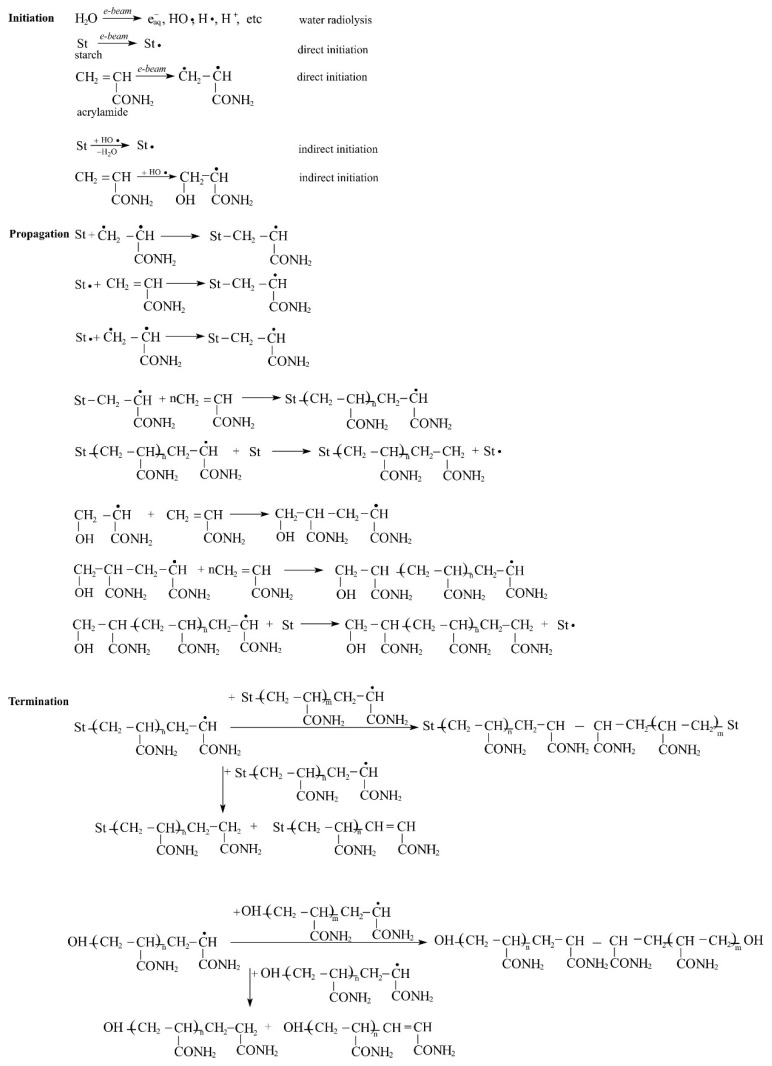
Mechanism of starch grafting by using electron beam irradiation.

The copolymerization mechanism of acrylamide onto starch by the exposure of an aqueous solution containing the monomer and polysaccharide substrate to the electron beam primarily involves the formation of free-radical sites in chemical reactants, initiated by an indirect effect due to water participating as a solvent. Nasef and Guven [54] stated that the accessibility of the monomer to the grafting sites and thus the participation in all steps of the grafting mechanism (initiation, propagation of the growing chain and termination) is practically affected by the diluting solvent. In our study, the solvent is water itself that absorbs most of the e-beam energy, leading to highly chemical reactive entities (i.e., $e_{aq}^{-}$, *H·*, *·OH*, $H_{2}O\cdot^{+}$, *H_2_*, O*) as a result of the primary effects of excitation, dissociation and ionization. Furthermore, the formed hydroxyl radicals attack the starch and acrylamide molecules generating their macroradicals, which then combine to produce the graft copolymer. Based on earlier literature [55,56,57,58,59], a possible mechanism of starch grafting in the radiation field is proposed in Scheme 2.

The copolymer-loaded silver nanoparticles (*Batch 3*) showed similar values (*p* > 0.5) of the conversion coefficient and residual monomer, respectively, as the non-loaded copolymer. Therefore, the addition of silver nanoparticles to the initial sample subjected to irradiation had no influence on the grafting level of the copolymer. The presence of silver in a metallic form does not have the ability to interfere in the initiation and termination steps of free-radical polymerization and thus it cannot affect the unreacted monomer amount. Recently, Velazco-Medel et al. [60] showed also that the presence of silver in the form of silver nitrate did not change the grafting yields for in the case of production of an antimicrobial silicone simultaneously grafted with acrylic acid and ethylene glycol dimethacrylate by using gamma radiation. However, it is possible that metallic silver rather participates in the design of the copolymer shape, which is not discernible by assessing the level of grafting. Thus, according to other previous reports [61,62], the presence of a large number of short or branched pAMD chains does not change the original compact shape of a polysaccharide to a greater extent, resulting in a lower hydrodynamic volume. Conversely, the formation of a small number of longer pAMD chains can modify the shape of a polysaccharide to a greater extent, retaining its larger hydrodynamic volume that is reflected in high intrinsic viscosity value.

### 3.2. Viscosity

Synthesized graft copolymers were soluble in cold water, unlike the native starch. It is well known that viscosity is strongly related to the molecular weight of polymers, influencing the functionality of the final product. The intrinsic viscosity is a measure widely used for the characterization of synthetic or natural polymers. On the other hand, the Huggins constant *k_H_* is considered an adequate criterion to evaluate the quality of the solvent, so that the higher the affinity between polymer and solvent, the lower is the *k_H_* value [63]. The closer its value is to 0.3, the higher is the affinity between polymer and solvent [64], indicating that the polymer presents a flexible and extended conformation and the polymer–solvent interactions are stronger than the polymer self-interactions (intra- and inter-molecular interactions) [63].

All graft copolymers had high intrinsic viscosity values ([*η*] > 7 dL/g) and *k_H_* below unity (Table 3). For copolymers synthesized by using a constant dose rate of 0.7 kGy/min (*Batch 1*), the highest value of the intrinsic viscosity was connected with the worst values of the conversion coefficient and residual monomer, respectively. Moreover, the intrinsic viscosity had a lower value as the irradiation dose increased than we expected. Several processes such as monomer polymerization, chain branching and cross-linking and degradation of the polymer already formed and even more competition among them can occur during irradiation, determining the final structure of the polymer in this way [65]. The cross-linking process was considered minimal, taking into account that the copolymers were water-soluble. Additionally, the reduction in the molecular weight of the polyacrylamide side chain was excluded due to the good results obtained for the monomer conversion coefficient and residual monomer content as the irradiation dose increased. Thus, the slight decrease in viscosity at a higher irradiation dose could be an effect of partial cleavage of the starch glycosidic linkages leading to a decrease in the molecular weight of the starch molecules when the irradiation dose increased. Additionally, one should also take into consideration the formation of a large number of shorter average chains of polyacrylamide that can lead to a lower molecular weight, although it is well known that high irradiation doses generally cause the degradation of starch and/or formed copolymer [31]. Similar findings were also reported for the preparation of chitosan-based flocculants with gamma irradiation-induced grafting [14], where at a dose greater than 0.6 kGy and a constant dose rate, the copolymer solution viscosity increased, and the monomer diffusion rate decreased accordingly, which led to a decrease in the grafting rate.

The copolymers prepared by using a constant irradiation dose of 1 kGy and varying the dose rate (*Batch 2*) showed better intrinsic viscosity values for dose rates of 0.6 and 0.7 kGy/min, respectively, exhibiting a good positive correlation between intrinsic viscosity and dose rate (*r*~0.721) in the range of investigated dose rate. These results were also correlated with the highest *C_c_* values and the lowest *R_m_* values.

The *k_H_* values for all synthesized copolymers were in the range of 0.2 to 0.7. Thus, the molecules of St-*g*-AMD 1, St-*g*-AMD 2 and St-*g*-AMD 4 interacted more with the solvent than with each other showing value of Huggins constant between 0.2 and 0.4, while some additional entanglement and intermolecular interaction were indicated by the Huggins constant for molecules of St-*g*-AMD 3 and St-*g*-AMD 5.

The copolymer-loaded silver nanoparticles (*Batch 3*) showed a significantly (*p* ≤ 0.05) higher value of the intrinsic viscosity (9.0 ± 0.2 dL/g) in comparison with the unloaded copolymer (7.4 ± 0.1dL/g). Thus, the addition of silver nanoparticles to the initial sample subjected to irradiation led to the formation of a copolymer with higher [*η*] and lower *k_H_*. These findings indicate that longer polyacrylamide (pAMD) chains are probably formed in the presence of AgNPs as a result of the intermolecular forces induced by metallic nanoparticles, thus obstructing the formation of short or branched chains of pAMD.

### 3.3. DSC Study

**Figure 1:**
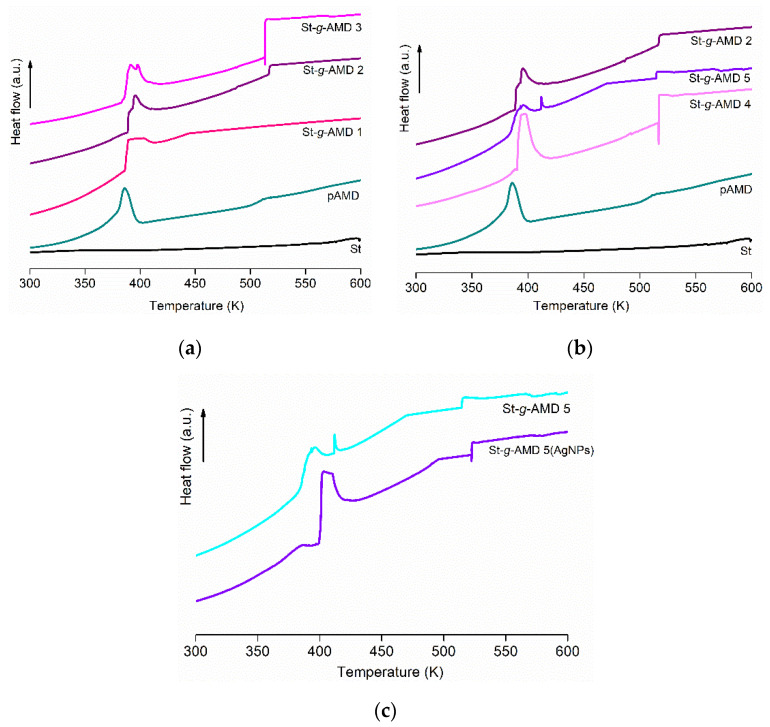
DSC curves of St, pAMD, and graft copolymers synthesized: (**a**) with the same $\dot{D}$ and different D (*Batch 1*), (**b**) with the same D and different $\dot{D}$ (*Batch 2*), and (**c**) with and without AgNPs (*Batch 3*).

DSC was used for determining the phase transitions (melting, glass transition) and decomposition of the synthesized copolymers in comparison with the native starch and polyacrylamide. The DSC curves of the investigated graft copolymers are displayed in Figure 1, and the values of thermodynamic parameters are presented in Table 4.

Two endothermic events could be observed in the DSC curve of native starch (St). The first endothermic peak (*Peak I*) located at around *T_I_* = 340 K can be attributed to the loss of absorbed moisture in the sample, while the second peak (*Peak II*) located around *T_II_* = 560 K corresponds to the crystalline melting of starch and thermal decomposition.

Polyacrylamide (pAMD) also showed two major events in the DSC curve: an endothermic peak (*Peak I*) at around *T_I_* = 385 K, which corresponds to the loss of absorbed moisture in the sample and the crystalline melting, and a glass transition at around *T_g_* = 506 K. It was reported that the degradation of pAMD occurs in the temperature range from 450 to 573 K as a result of the liberation of ammonia (volatile product) and formation of imide groups in the cyclization phenomenon [66,67]. Different temperatures at lower values (466–472 K) of glass transition were also reported previously in the literature for pAMD [66,68].

The graft copolymers presented thermal events characteristic to the St and pAMD shifted to higher temperatures in the DSC curves. For copolymers prepared by using a constant $\dot{D}$ = 0.7 kGy/min (*Batch 1*), the DSC curves (Figure 1a) showed endothermic peaks shifted to higher temperature values (*p* ≤ 0.05) in comparison with pAMD and St. Moreover, molecules of St-*g*-AMD 2 and St-*g*-AMD 3 showed that *Peak I* consisted of two overlapped peaks (*T_I_*~387 K and T_I_ *~393 K). On the other hand, the second peak, typical for crystalline melting of polysaccharides, could not be identified for any samples of this batch. The glass transition could also not be detected for the copolymer synthesized by using the smallest dose irradiation (St-*g*-AMD 1), whereas the other copolymers showed the increase (*p* ≤ 0.05) of *T_g_* value in comparison with pAMD (Table 4). Worzakowska [69] reported also shifts of *T_g_* to higher temperature values for other starch-based copolymers in comparison with the *T_g_* of homopolymer as a result of the lower chain mobility of the newly synthesized copolymers. The results indicated that the temperature *T_I_* had a relatively good positive correlation (*r*~0.759) with the irradiation dose D and very good correlations with the grafting parameters, namely the conversion coefficient *C_c_* (*r*~0.999) and the residual monomer *R_m_* (*r*~−0.997), respectively, and a good correlation (*r*~0.831) with the intrinsic characteristic copolymer, namely the viscosity (*η*). At the same time, the process enthalpy *ΔH_I_* was very well correlated (*r*~0.965) with the irradiation dose D and only poorly correlated with the grafting parameters: *C_c_* (*r*~0.599) and *R_m_* (*r*~−0.620).

Changes in the temperatures of the thermal events were also observed for the copolymers synthesized by using a constant irradiation dose of 1 kGy and varying the dose rate (*Batch 2*) compared to the native starch and pAMD. The copolymers St-*g*-AMD 4 and St-*g*-AMD 5 obtained by irradiation at 0.5 and 0.6 kGy/min, respectively, displayed two endothermic peaks in a similar manner (Figure 1b), with *T_II_* shifting to lower values in comparison to a starch macromolecule (Table 4). For all samples, the values of *T_g_* showed an increase (*p* ≤ 0.05) in comparison with the *T_g_* of homopolymer. At the same time, a reduction (*p* ≤ 0.05) of the *T_g_* values was observed and it was very well correlated (*r* = 0.921) with the increase in the dose rate $\dot{D}$. For this batch, the results indicated that the temperature *T_I_* had very good correlations with the dose rate $\dot{D}$ (*r* = 0.998), as well as with the grafting parameters, namely *C_c_* (*r* = 0.981) and *R_m_* (*r*~−0.985), respectively, and only poor correlation (*r*~0.677) with the copolymer intrinsic viscosity (*η*). Moreover, the process enthalpy *ΔH_I_* was very well correlated with the dose rate $\dot{D}$ (*r* = 0.992), *C_c_* (*r* = 0.985) and *R_m_* (*r* = −0.988) and poorly correlated with the intrinsic viscosity (*η*) (*r*~0.693).

The DSC curves of the silver-loaded and unloaded copolymers were apparently similar (Figure 1c). The copolymer-loaded silver nanoparticles (*Batch 3*) showed significantly (*p* ≤ 0.05) lower values of peak temperatures while the *T_g_* had a higher value (*p* ≤ 0.05) in comparison with the unloaded copolymer (Table 4). This finding indicates that the addition of silver nanoparticles to a copolymer led to a final product (silver-loaded copolymer) with a lower melting temperature and higher value of *T_g_*.

Consequently, the graft copolymers synthesized in this work had both higher temperatures attributed to the loss of absorbed moisture than the polysaccharide (St) and higher glass transition temperatures than the polyacrylamide (pAMD). In other words, we can consider that, in terms of the glass transition temperature, the graft copolymers showed better thermal stability than the homopolymer (pAMD). Other reports also revealed that natural polysaccharides grafted with vinyl monomer by means of ionizing radiation had good thermal stability in the field of practical applicability [47,70,71]. Moreover, da Silva et al. [33] clearly proved that grafting of pAMD chains onto the polysaccharides enhances their thermal stability.

### 3.4. Flocculation Investigation

The functionality of the graft copolymers as flocculant agents was examined on surface water collected from the meat processing industry.

The quality parameters measured were pH, total suspended solids (*TSS*), fatty matters (*FM*), chemical oxygen demand (*COD*), and biochemical oxygen demand (*BOD_5_*). The characteristics of raw water and the permissible levels of water quality indicators according to the Romanian national guideline are shown in Table 5.

**Figure 2:**
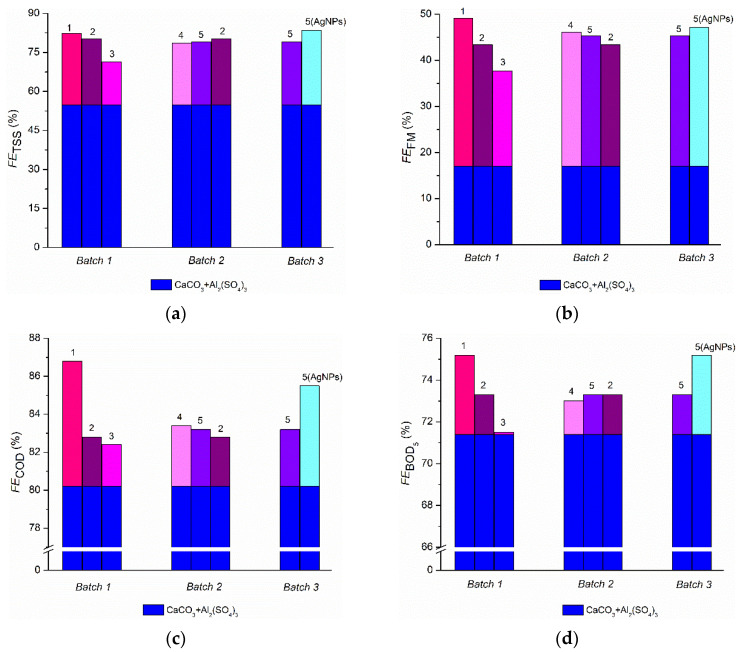
Results of the flocculation study at laboratory level: (**a**) total suspended solids (*TSS*), (**b**) organic matters (*FM*), (**c**) chemical oxygen demand (*COD*), and (**d**) biochemical oxygen demand (*BOD_5_*). The coagulation–flocculation experiments involved inorganic coagulants (CaCO_3_ and Al_2_(SO_4_)_3_–blue color) and aqueous solution flocculant (graft copolymer).

Flocculation performances of the inorganic coagulants and their combination with a flocculant (graft copolymer) are presented in Figure 2.

All synthesized copolymers showed good performances in the coagulation–flocculation process. The pH value of raw water decreased significantly after treatment with inorganic coagulants and then slowly from 7.1 to the value of 6.9 by adding flocculant to the inorganic coagulants in the water treatment. The copolymer presence increased the yield of the total suspended solids from 55% for inorganic coagulants to approximately 80%. A *TSS* yield of 83.5% was reached by using St-*g*-AMD 5(AgNPs) copolymer in the coagulation–flocculation process. The yield of the fatty matters reached 17% when only inorganic coagulants were used and increased to approximately 45% by adding the synthesized copolymers. The copolymers St-*g*-AMD 1 and St-*g*-AMD 5(AgNPs) showed the best values of *FM* yield. The yields for the other investigated indicators (*COD* and *BOD_5_*) were improved only slightly by adding copolymers to the inorganic coagulants in the water treatment.

The elimination of suspended solids and as much organic material as possible is an essential trait in the process of wastewater flocculation [72]. Therefore, our graft copolymers exhibited propitious flocculating capacity by reducing investigated environmental parameters (*TSS*, *FM*, *COD* and *BOD_5_*) of water collected from a meat processing plant. Among the graft copolymers, the copolymer-loaded silver nanoparticles St-*g*-AMD 5(AgNPs) showed superior results by significantly reducing the total organic load (*TSS*, *FM*, *COD* and *BOD_5_*) of the tested water.

## 4. Conclusions

“Green” starch-graft copolymers with eco-friendly approaches were successfully synthesized via graft copolymerization in aqueous solutions of acrylamide (AMD) onto a starch substrate (St) induced by an electron beam irradiation. Therefore, for the copolymers prepared in the experimental conditions applied herein, the main findings can be summarized as follows:

1. The monomer conversion coefficient *C_c_* exceeded 90%, while the residual monomer *R_m_* had values below 3%. However, these grafting parameters were much better correlated with dose rate $\dot{D}$ than irradiation dose D.
2. All graft copolymers had high intrinsic viscosity values ((*η*) > 7 dL/g) and Huggins constant *k_H_* below unity.
3. The addition of AgNPs to the initial sample subjected to irradiation had no influence on the grafting level but led to the formation of copolymer with higher intrinsic viscosity and a lower Huggins constant.
4. The graft copolymers presented thermal events characteristic to the native starch (St) and polyacrylamide (pAMD) shifted to higher temperatures in the DSC curves. The identified changes confirmed the grafting process and formation of new chemical bonds between the St backbone and pAMD. At the same time, all graft copolymers showed improved thermal stability in terms of glass transition temperatures higher than the polyacrylamide.
5. The potential of the synthesized copolymers to improve the quality of the surface water from the meat industry was proven in coagulation–flocculation experiments at the laboratory level. Thus, the copolymers were able to reduce the water quality indicators, such as total suspended solids, fatty matters in suspension, chemical and biochemical oxygen demands.

Therefore, the characteristics of the starch-based flocculants synthesized via graft copolymerization induced by an electron beam irradiation depend on the irradiation parameters, namely the irradiation dose D and dose rate $\dot{D}$. These parameters need to be carefully investigated whenever the synthesis of flocculants with certain intrinsic viscosity values or efficiency in a certain application is desired.

## Figures and Tables

**Table 1:** Materials used for radiation-induced graft copolymers synthesis.

Substance	Chemical Formula	Chemical Properties
Starch (St)	(C_6_H_10_O_5_)*_n_*	Amylose content: ~27% Amylopectin content: ~73% Moisture content: 12%
Acrylamide (AMD)	CH_2_=CHCONH_2_ or C_3_H_5_NO	Molecular weight: 71.08 g/mol Density: 1.322 g/cm^3^ Boiling point: 125 °C/25mm Melting point: 82–85 °C Flash point: 138 °C
Silver nitrate (used for preparation of silver nanoparticles)	AgNO_3_	Molecular weight: 169.86g/mol Melting point: 212 °C
Sodium chloride	NaCl	Molecular weight: 58.44 g/mol Density: 2.165 g/cm^3^ Boiling point: 1413 °C Melting point: 801 °C

**Table 2:** Synthetic details of the irradiations and sample marking.

Sample	Irradiation Dose, D (kGy)	$\mathrm{Dose}\;\mathrm{Rate},\;\dot{D}$ (kGy/min)
***Batch 1***		
St-*g*-AMD 1	0.7	0.7
St-*g*-AMD 2	1.0	0.7
St-*g*-AMD 3	1.2	0.7
***Batch 2***		
St-*g*-AMD 4	1.0	0.5
St-*g*-AMD 5	1.0	0.6
St-*g*-AMD 2	1.0	0.7
***Batch 3***		
St-*g*-AMD 5	1.0	0.6
St-*g*-AMD 5 (AgNPs)	1.0	0.6

**Table 3:** Grafting and rheological parameters for graft copolymers.

Sample	D (kGy)	$\dot{D}$ (kGy/min)	*C_c_* (g/100g)	*R_m_* (g/100g)	[*η*] (dL/g)	*k_H_*
***Batch 1***						
St-*g*-AMD 1	0.7	0.7	91.6 ± 0.3	2.8 ± 0.1	7.9 ± 0.1	0.18
St-*g*-AMD 2	1.0	0.7	96.6 ± 0.5	1.1 ± 0.2	7.3 ± 0.0	0.33
St-*g*-AMD 3	1.2	0.7	95.3 ± 0.3	1.5 ± 0.1	7.8 ± 0.6	0.71
***Batch 2***						
St-*g*-AMD 4	1.0	0.5	95.3 ± 0.4	1.6 ± 0.1	7.0 ± 0.2	0.37
St-*g*-AMD 5	1.0	0.6	96.1 ± 0.4	1.3 ± 0.1	7.4 ± 0.1	0.72
St-*g*-AMD 2	1.0	0.7	96.6 ± 0.5	1.1 ± 0.2	7.3 ± 0.0	0.33
***Batch 3***						
St-*g*-AMD 5	1.0	0.6	96.1 ± 0.4	1.3 ± 0.1	7.4 ± 0.1	0.72
St-*g*-AMD 5 (AgNPs)	1.0	0.6	96.5 ± 0.3	1.2 ± 0.1	9.0 ± 0.2	0.19

**Table 4:** Thermodynamic parameters of graft copolymers.

Sample	*Peak I*			*Peak II*		Glass Transition	
	*T_I_* (K)	T_I_* (K)	Δ*H_I_* (J/g)	*T_II_* (K)	Δ*H_II_* (J/g)	*T_g_* (K)	Δ*C_p_* (J/g K)
St	339.70 ± 0.41	-	248.77 ± 1.76	560.86 ± 1.55	-	-	-
pAMD	384.87 ± 0.46	-	681.33 ± 1.00	-	-	505.94 ± 0.83	5.23 ± 0.44
***Batch 1***							
St-*g*-AMD 1	386.53 ± 0.54	-	337.20 ± 0.74	-	-	-	-
St-*g*-AMD 2	387.88 ± 0.78	393.53 ± 0.63	412.73 ± 1.34	-	-	514.84 ± 0.52	11.23 ± 1.12
St-*g*-AMD 3	387.47 ± 0.87	391.15 ± 0.32	542.73 ± 0.95	-	-	516.25 ± 0.74	43.30 ± 0.44
***Batch 2***							
St-*g*-AMD 4	384.30 ± 0.41	-	4.94 ± 0.53	390.12 ± 0.35	514.27 ± 0.69	516.18 ± 0.30	23.62 ± 0.38
St-*g*-AMD 5	385.90 ± 0.66	-	194.99 ± 0.38	411.21 ± 0.45	18.24 ± 0.38	515.02 ± 0.36	7.89 ± 0.34
St-*g*-AMD 2	387.88 ± 0.78	393.53 ± 0.63	412.73 ± 1.34	-	-	514.84 ± 0.52	11.23 ± 1.12
***Batch 3***							
St-g-AMD 5	385.90 ± 0.66	-	194.99 ± 0.38	411.21 ± 0.45	18.24 ± 0.38	515.02 ± 0.36	7.89 ± 0.34
St-*g*-AMD 5 (AgNPs)	371.94 ± 0.43	-	44.90 ± 0.17	400.33 ± 0.56	381.68 ± 0.41	522.03 ± 0.47	5.80 ± 0.38

**Table 5:** Characteristics of the raw water used in experiments.

Parameter	Raw Water	Maximum Allowed Level *
*pH*	8.5	6.5–8.5
*TSS* (mg/L)	182	35
*FM* (mg/L)	106	20
*COD* (mg O_2_/L)	227	125
*BOD_5_* (mg O_2_/L)	105	25

* Romanian National Regulation NTPA 001/2005–Limits of pollutant loadings for industrial and municipal wastewaters at discharge in natural receivers.

## Data Availability

Data sharing is not applicable for this article.
